# Mid- and Long-Term Results Using 448 kHz Stimulation on the Elasticity of the Supraspinatus Tendon Measured by Quantitative Ultrasound Elastographyin Badminton Professionals: Prospective Randomized Double-Blinded Clinical Trial with Nine Months of Follow-Up

**DOI:** 10.3390/jcm11061664

**Published:** 2022-03-17

**Authors:** Santiago Navarro-Ledesma, Ana Gonzalez-Muñoz

**Affiliations:** 1 Department of Physiotherapy, Campus of Melilla, University of Granada, Querol Street 5, 52004 Melilla, Spain; 2 Clinica Ana Gonzalez, 29018 Malaga, Spain; anagonzalez.fisioterapueta@gmail.com

**Keywords:** hyperthermia, radiofrequency, supraspinatus tendon, ultrasound, elastography, badminton

## Abstract

The aim of this study is to analyse the changes that occur in the elasticity of the supraspinatus tendon after the application of a 448 kHz capacitive resistive monopolar radiofrequency (CRMR) at 3, 6 and 9 months in professional badminton players. A randomized double-blinded clinical trial that included 9 months of follow-up was used. A private care practice was used to recruit the participants of this study. They were randomly assigned either the CRMR treatment (*n* = 19) or the placebo treatment (*n* = 19). The experimental group received a total of nine treatments of 448 kHz CRMR divided into three treatments per week. The control group received the same regimen but with no radiofrequency. Quantitative ultrasound strain elastography was used to report the main values for three areas of the supraspinatus tendon. These were measured at the start (T1) and directly after (T2), one week after, (T3), three months after (T4), six months after (T5) and nine months after (T6) the completion of the intervention program. There were statistically significant differences in the supraspinatus tendon elasticity immediately after (*p* ≤ 0.001), one week after (*p* ≤ 0.001) and three months after (*p* = 0.01) the intervention program. No significant changes were found six or nine months after the intervention program. A three-week intervention program using 448 kHz produced significant changes in the elasticity of the supraspinatus tendon, with the changes lasting up to approximately three months when compared to the control group.

## 1. Introduction

Capacitive resistive monopolar radiofrequency (CRMR) has been widely used to treat different musculoskeletal disorders, such as osteoarthritis of the knee [1], cervico-brachial pain [2] and low back pain [3]. Furthermore, a CRMR application has shown other effects, such as a greater effect on haemoglobin saturation [4], immunological responses [5,6] and changes in tendon elasticity but only in the short term [7]. In this regard, hyperthermia techniques appear to be promising as a way to increase vascularity; blood perfusion; temperature; and therefore, the viscoelasticity of the soft tissue, which is thought to be crucial in keeping the musculoskeletal system healthy and functional [7,8,9]. Thus, it is important to research techniques that try to reach this goal and to assess their effects on the tissue over time. However, the use of ultrasound elastography to assess how CRMR affects tendon elasticity has only been evaluated in the short term, and a gap still remains as to its extent in the medium and long terms.

The stiffness of the area in question as well as the tissues surrounding it can be defined by using strain elastography (SEL) [10,11]. This allows the mechanical properties of the tendon to be evaluated, which goes beyond the scope of the macroscopic assessment [12]. The Young modulus (Y: ¼ stress/strain) is used to express the elasticity of the tissue, in which stress is the externally applied force and strain is the internal response in the form of tissue deformation Y. Since there are no direct measurements of stress using SEL, it is hypothesized that presenting a ratio between the area of interest and a reference area (provided both areas are subjected to the same amount of manual pressure, i.e., the same tissue depth) will produce the most reliable results [1]. With regard to shoulder conditions, significant correlations of clinical tests and questionnaires such as VAS, Quick DASH, Constant–Murley score, Simple Shoulder Test, ASES score and UCLA score have been found in relation to SEL as well as to MRI in patients with supraspinatus tendinopathy [13,14] and supraspinatus tendon tears [15].

The validity of SEL in the supraspinatus tendon has been shown [13,14,15,16,17], as has its ability to detect changes in the elasticity of both the supraspinatus tendon and muscle in participants who are healthy [7,16]. Current research shows the supraspinatus tendon elasticity to be around 3.75 in healthy subjects, 3.8 in healthy sports populations and 3.55 in subjects with supraspinatus tendinopathy [7,17,18,19]. To date, laboratory studies have shown promising effects, but there is an important need to increase knowledge about these interventions in order to extrapolate results to clinical settings. In this regard, CRMR appears to be a potential intervention in modulating the elastic properties of soft tissue, and its effects on immunological responses can also be hypothesized. However, no studies exist that analyse either the changes in the elasticity of the supraspinatus tendon in the medium and long terms after a CRMR intervention or the clinical extrapolations from the results obtained.

Therefore, the aim of the study is to analyse the changes that occur in the elasticity of the supraspinatus tendon after the application of a 448 kHz capacitive resistive monopolar radiofrequency (CRMR) at 3, 6 and 9 months in professional badminton players. This will help in the understanding of which response is expected to occur under physiological and healthy circumstances after the use of a CRMR intervention. Once this first step has been accomplished, the results can be extrapolated in order to plan injury prevention programs and treatments for those with shoulder conditions as well as to strengthen the use of SEL as a tissue-quality measurement tool. In addition, these results may also serve as a first step toward new research, not only in the effects produced in shoulder tendons but also in other tendons with different characteristics, in sports populations as well as others.

## 2. Material and Methods

### 2.1. Design

A placebo controlled, randomized, double-blinded clinical trial that included 9 months of follow-up was used.

### 2.2. Setting

A private care practice in Malaga, Spain, was used to recruit the subjects. Informed written and verbal consent were obtained from all participants before enrolment, and baseline demographic and clinical data were collected. Participants were informed of the trial through formal meetings and trial information sheets. The study was registered in ClinicalTrials.gov (Clinical trial registration: NCT04273633, Clinicaltrial.gov. Registered 18 September 2020, https://www.clinicaltrials.gov/ct2/show/NCT04273633) and conducted in accordance with the Declaration of Helsinki. A Medical Research Ethics Committee (984/CEIH/2019) approved the study, following the CONSORT Statement [20,21].

### 2.3. Participants

A screening process was used to determine the suitability of participants.

The criteria used to include participants were as follows: participants (i) 18 to 64 years of age; (ii) national level badminton players in good health; and (iii) no shoulder injuries or shoulder pain during the previous year.

The criteria used to exclude participants were defined as follows: participants presenting any (i) painful and inflammatory process, or (ii) neurological or (iii) orthopaedic diseases that can alter balance, hearing, vision or cognitive impairment and that might impact their ability to answer questions.

### 2.4. Allocation

A random number generator controlled by a research assistant was used to randomize participants.

During the intervention period, the participants were not allowed to receive other treatments or to participate in any other regimen or study.

The allocation of the subjects was blinded to the participants and the statistician.

An experienced physiotherapist who collaborated in the research carried out all the interventions.

The Template for Intervention Description and Replication (TIDieR) Checklist recommendations was followed [19].

### 2.5. Sample Size Calculation

The EPIDAT program, which was used to determine the sample size, is a free distribution program developed by public institutions and aimed at epidemiologists and other health professionals for data management. Based on previous studies, using an average of 1.1 mm as the expected supraspinatus mean difference and the corresponding standard deviation of 1.0 mm with significance set at α = 0.05, a power analysis for t tests indicated a sample size of *n* = 19 per group for 90% power [22,23,24].

### 2.6. Intervention Description

#### 2.6.1. Experimental Group

Participants received a total of nine 448 kHz CRMR interventions, with three interventions per week for three weeks. The patient was asked to lie laterally with their desired shoulder pointing up [8,25]. The INDIBA^®^ Activ 8 equipment was used with a peak power of 200 W and 450 VA, and a 448 kHz CRMR was applied. Capacitive (CAP) and Resistive (RES) waves were applied through a coupling medium using electrodes made of metal. The patient’s perception was expected to be 8 out of 10; therefore, they let the therapist know when that perception was felt, so that both CAP and RES modes were delivered as a thermal dose following manufacturer guidelines. A total of 20 min for shoulder intervention was used, with both CAP and RES modes being delivered for 10 min. The patient held the return electrode on the side that corresponded with the treated shoulder.

#### 2.6.2. Control Group

Participants in the control group received a quantity of interventions and duration times corresponding to those of the experimental group. The intervention was manually carried out using only the electrodes but without any active current. All participants continued their normal training and had the same number of sessions per week until the end of the study once the intervention program was finished.

### 2.7. SEL Measurements

A physiotherapist with 10 years of experience using ultrasound images of the musculoskeletal system used a Logiq S7 with a 15 MHz linear probe (GE Healthcare, Milwaukee, WI, USA) to carry out all measurements. The measurements were performed following recommendations from previous studies, with the patient sitting in an erect posture with the arm internally rotated, the palm placed over their iliac wing or “back pocket”, and the elbow flexed and directed medially [26], and a transverse glide was then carried out at the site to determine the exact position where the assessor evaluated the tendon thickness to be at its maximum. Supraspinatus tendon measurements were taken 2 cm laterally from the bicep tendon, where it is usually assessed [27,28]. Then, the skin was compressed from 2 to 5 mm [11]. A software-incorporated quality control consisting of a display of green bars, one bar being the least acceptable and five bars the most acceptable, was used to evaluate the recommended compression size. As indicated by the manufacturer guidelines and following previous studies [17], a 5 mm circular region in a soft part of the area of interest was used to calculate the exact raw strain value. Values were shown from 0 to 6, the softest shown as 0 and the hardest as 6.

To evaluate the tendon, three different measurement points were used. In order to minimise intra-observer variation, the mean at each of these three areas points was calculated. Sequences that resulted in green bars were the only ones included, meaning that the images were of the highest quality. This followed the manufacturer’s recommendations.

### 2.8. Statistical Analysis

SPSS^®^ Statistics version 21.0 (IBM, Chicago, IL, USA) was used for all analyses. The Shapiro–Wilk test was used to verify data distribution normality. To compare the two groups (CRMR intervention and control groups) at baseline and follow-ups regarding clinical characteristics, a six-way repeated measures ANOVA was conducted, with six levels corresponding to each time of assessment (T1, T2, T3, T4, T5 and T6), and the two intervention groups as independent factors. A *p*-value < 0.05 was considered statistically significant. Bonferroni adjustments for multiple comparisons were used. The differences between group effect sizes, for all quantitative variables, were measured using the Cohen d coefficient. The effect sizes that were greater than 0.8 were considered large, those around 0.5 were considered moderate and those less than 0.2 were considered small.

## 3. Results

**Figure 1:**
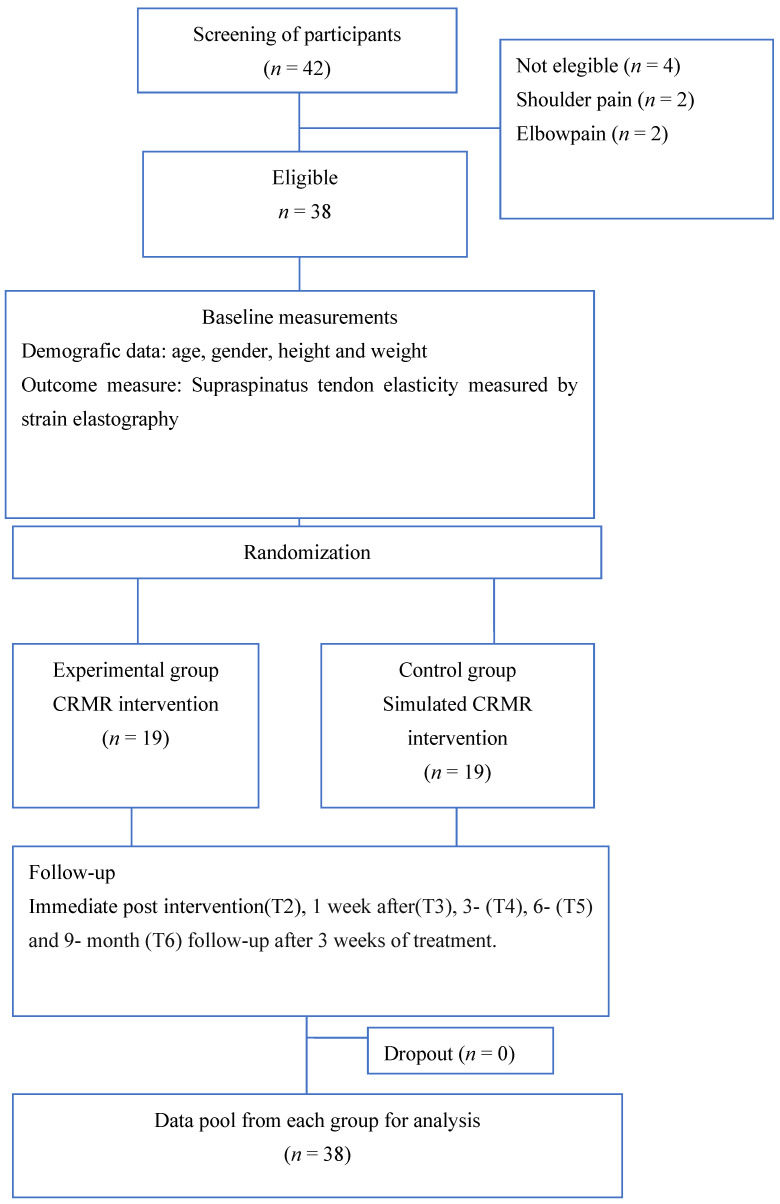
Flow diagram of participants.

A total of 42 participants were recruited, with eight participants who did not meet the inclusion criteria, four of them because of shoulder (*n* = 2) and elbow pain (*n* = 2). Finally, a total of 38 participants were enrolled in the experimental (*n* = 19) and the control group (*n* = 19) and completed the baseline assessment. Quantitative SEL was used to assess all participants before the treatment (T1) and immediately (T2), seven days (T3), three months (T4), six months (T5) and nine months (T6) after the completion of the intervention program (Figure 1). The study reached its goal and ended subsequent to the recruitment of the necessary sample size and once comparisons between groups before and after the intervention were analysed.

The experimental group presented dermatological responses such as flushing due to the hyperthermia used during treatment, whereas the control group did not present any response and was not thought to be damaged because of the sham intervention with no temperature change. None of the participants suffered any harm during the study. Formal meetings and trial information sheets were used to inform participants.

### 3.1. Sample Characteristics

Table 1 shows demographic characteristics. In terms of age, gender, height, weight and supraspinatus tendon elasticity, there were no significant differences.

### 3.2. Differences on Supraspinatus Tendon Elasticity between Groups

Table 2 describes the comparisons between groups. Statistically significant differences in the supraspinatus tendon elastic properties were presented immediately after (T2), seven days after (T3) and three months after (T4) the treatment ended. There were no significant differences at six months (T5) and nine months (T6) after its end.

## 4. Discussion

Differences in the supraspinatus tendon elasticity were presented when comparing groups immediately after (T2), seven days after (T3) and three months after (T4) the intervention. No significant changes were found when assessing six- (T5) and nine- (T6) month follow-ups.

The results from the present study show that the effects of CRMR on the supraspinatus tendon elasticity may last up to three months in a healthy sports population after a 3-week treatment program, when compared to controls. These findings are a first step to understanding the duration of the CRMR effects in the tissue and therefore how long the benefit may last. In this regard, the main CRMR effects postulated have been cell proliferation, collagen remodelation, increased quality of the tissue [29,30,31,32,33] and immunological responses [5,6,32], through the increase in other responses such temperature and viscoelastic changes, and blood perfusion, thus keeping the tissue healthy and preventing it from degeneration [4].

Furthermore, the addition of daily exercise may be part of the increase in the lasting effects of CRMR on the tissue. Nevertheless, the main factor to explain differences between groups should be attributed to the use of CRMR during the three-week treatment program, given that the same amount of exercise was carried out in both groups during the entire research project.

To date, only short-term effects of CRMR on tendon tissue assessed by elastography have been described. The current study presents an analysis of such effects up to nine months after treatment and hence is the first in this regard; therefore, comparison to other studies is difficult. Previous studies have shown supraspinatus tendon properties and tendon assessment by elastography in healthy populations [13,14,15,16,17], presenting results that are in line with ours [17,18]. Differences in elastic properties of the tendon values are explained by blood perfusion and the consequent increase in temperature, with stiffer tissues presenting higher values on SEL measurement and softer tissues lower values on SEL measurement [9,12,34]. In this context, SEL supraspinatus mean values are known to be around 3.55 in subjects with supraspinatus tendinopathy, 3.75 in healthy subjects and 3.8 in a healthy sports population [7]. Differences between studies are explained by age and sex [17,18,19].

On the other hand, changes in supraspinatus SEL in the short term have also been studied after 12 weeks of unilateral shoulder exercises, showing no significant differences when comparing patients with supraspinatus tendinopathy and controls [19]. Therefore, differences in tendon elasticity may be attributed to the use of CRMR when compared to our results, indicating that the addition of a 3-week CRMR intervention program to a sports population’s usual training may be a key factor in producing viscoelastic changes measured by SEL.

## 5. Strengths and Weaknesses

The present study shows a number of strengths, since it is a first step toward better understanding the effects of a 3-week CRMR treatment program on supraspinatus SEL in a healthy sports population with a nine-month follow-up. Demographic characteristics were similar in both groups. Furthermore, all SEL measurements were carried out by an expert in musculoskeletal ultrasound imaging, ensuring the high quality of the values obtained. Finally, given that the vascularity of the subacromial bursa and rotator cuff tendons is a key factor in the pathogenesis of subacromial bursitis and impingement syndrome, rotator cuff tendinitis and rotator cuff tears, which alter the ratio between subacromial space and supraspinatus tendon [35], we decided that the patient should lie laterally with the shoulder of interest on the upper side, where the main vascular and neural structures were stimulated [25]. However, the present study has some limitations that should be recognized. First, the person who carried out the treatment was aware of whether the subject was from the control or the experimental group, so triple-blinding was not achieved. Second, although the person measuring the supraspinatus elasticity was an expert and the ultrasound equipment incorporated a tool to ensure the quality of the measurements, ultrasound imaging is examiner-dependent, and this should be considered when interpreting the findings. Third, this study was carried out in healthy players, so the extrapolation of findings to other populations should be made with caution. Finally, participants were instructed not to talk about the treatment with any other participant until the whole research project was completed; nevertheless, we cannot guarantee that this was achieved, and therefore, blinding could be affected in any case.

## 6. Clinical Implications

The results of the present study show clinical relevance. The preservation of the CRMR effects up to three months may let the subjects benefit from longer responses with better adaptation to both loads and immunological responses; therefore, it may modulate inflammation when potential injuries or repetitive activities occur. Furthermore, assessing changes in the elasticity of the tissue over time may help clinicians detect possible injuries as well as serve as an indicator of recovery. These findings may elucidate new treatment lines for those suffering from supraspinatus tendinopathy. However, this is the first long-term study and more research is needed in this line. In addition, studying SEL changes after a CRMR treatment in both loading and non-loading tendons is necessary, not only in healthy subjects but also in those suffering from different conditions. Furthermore, studies assessing changes in SEL after the use of CRMR in combination with exercise or CRMR alone should be carried out. Finally, given that immunological responses after CRMR have been shown, we think that this should be further assessed, especially which kind of immune cells are present during such a response. In this regard, stating a difference between the type of macrophages (m1 or m2) as well as the type of interleukins (IL1-IL6 or IL4-L10) that are present would indicate whether the neuro–endocrine–immune pathways used lead to an efficient anti-inflammatory response. This is explained by the stimulation of stem cells after using hyperthermia, which directly intervenes in the control of the inflammatory processes by secreting anti-inflammatory interleukins [2,3,4,5].

## 7. Conclusions

The use of a 448 kHz stimulation program induced changes in the elastic properties of the supraspinatus tendon measured by SEL, which can last approximately for three months. These findings are a first step toward helping us understand how CRMR effects may last in the tissue and therefore may help us when planning injury prevention, improving performance, or developing a treatment program, through improved adaptations of the tendon to both loads and immunological responses. Further studies assessing changes in the supraspinatus elasticity as well as in immune and neuro–endocrine pathways are needed to fully explain the presented results.

## 8. Key Points

Findings: For the first time, the changes that occurred in the elasticity of the supraspinatus tendon after a 448 kHz CRMR at 3, 6 and 9 months are shown.

The effects of capacitive resistive monopolar radiofrequency on the supraspinatus tendon elasticity have been shown to last up to approximately three months.

Implications: The presented findings permit the behaviour of supraspinatus tendon elasticity after a CRMR intervention to be known, and this will open new possibilities in both optimisation and treatments in soft tissue in general and particularly in supraspinatus tendon in a sports population. Furthermore, using a CRMR intervention leads to responses such as cell proliferation, collagen remodelation, increased quality of the tissue, increase in blood perfusion, and temperature and viscoelastic changes, which may prevent the tissue from degenerating and may keep it healthy through a better adaptation to both loads and immunological responses when potential injuries or repetitive activities occur.

Cautions: This study was carried out in healthy players, so the extrapolation of findings to other populations should be made with caution.

## Figures and Tables

**Table 1:** Baseline demographic characteristics.

	Intervention Group (CRMR) (*n* = 19)	Control Group (Placebo CRMR) (*n* = 19)
Age (years), mean (SD)	41.6 (13.7)	43.8 (10.6)
Height (cm), mean (SD)	1.72 (0.8)	1.73 (0.8)
Weight (kg), mean (SD)	72.6 (12.1)	73.5 (11.3)
Supraspinatus tendon elasticity, mean (SD)	3.88 (0.47)	3.79 (0.57)

CRMR: Capacitive resistive monopolar radiofrequency. SD: Standard Deviation.

**Table 2:** Between group differences on supraspinatus tendon elasticity at baseline; after the intervention (T2); and at one-week (T3), three-month (T4), six-month (T5) and nine-month (T6) follow-ups (95% CI).

	T1 (Baseline)	T2 (Immediately after the CRMR Intervention)	T3 (One-Week Follow-Up)	T4 (Three-Month Follow-Up)	T5 (Six-Month Follow-Up)	T6 (Nine-Month Follow-Up)
Supraspinatus tendon elasticity (mean)	0.09 *p* = 0.59 (−0.25; 0.44) ^a^	−1.00 *p ≤* 0.001 ** (−1.41; −0.59) ^a^	−0.64 *p ≤* 0.001 ** (−0.98; −0.31) ^a^	−0.41 *p* = 0.01 * (−0.73; −0.09)	−0.01 *p* = 0.94 (−0.34; 0.32)	0.13 *p* = 0.44 (−0.20; 0.26)
Cohen’s d	0.17	−1.62	−1.25	−0.84	−0.02	0.26
SE difference	0.17	0.20	0.16	0.15	0.16	0.16

* Statistically significant differences (*p* = 0.05). ** = *p* < 0.001. SE: Size effect. ^a^ 95% CI.

## Data Availability

Not applicable.
